# A novel pyroptosis-related gene signature exhibits distinct immune cells infiltration landscape in Wilms’ tumor

**DOI:** 10.1186/s12887-024-04731-0

**Published:** 2024-04-27

**Authors:** Yujun Guo, Wenjun Lu, Ze’nan Zhang, Hengchen Liu, Aodan Zhang, Tingting Zhang, Yang Wu, Xiangqi Li, Shulong Yang, Qingbo Cui, Zhaozhu Li

**Affiliations:** 1 Department of Pediatric Surgery, The Sixth Affiliated Hospital of Harbin Medical University, Harbin Medical University, No.998 Aiying Street, Harbin, Heilongjiang 150027 China; 2 Key Laboratory of Growth Regulation and Translational Research of Zhejiang Province, School of Life Sciences, Westlake University, Hangzhou, Zhejiang 310024 China; 3 Center for Infectious Disease Research, Westlake Laboratory of Life Sciences and Biomedicine, Hangzhou, Zhejiang 310024 China; 4 Laboratory of Systems Immunology, Institute of Basic Medical Sciences, Westlake Institute for Advanced Study, Hangzhou, Zhejiang 310024 China; 5 Department of Colorectal Surgery and Oncology (Key Laboratory of Cancer Prevention and Intervention, China National Ministry of Education, Key Laboratory of Molecular Biology in Medical Sciences, Zhejiang Province, China), The Second Affiliated Hospital of Zhejiang University School of Medicine, No.88 Jiefang Road, Hangzhou, Zhejiang 310022 China; 6 Department of Pediatric Surgery, The Second Affiliated Hospital of Harbin Medical University, Harbin Medical University, No.246 Xuefu Road, Harbin, Heilongjiang 150000 China; 7 Psychology and Health Management Center, Harbin Medical University, No.157 Baojian Road, Harbin, Heilongjiang 150081 China

**Keywords:** Wilms’ tumor, Pyroptosis, Immune infiltration, Bulk RNA-seq, Single-nuclear RNA-seq

## Abstract

**Background:**

Wilms’ tumor (WT) is the most common renal tumor in childhood. Pyroptosis, a type of inflammation-characterized and immune-related programmed cell death, has been extensively studied in multiple tumors. In the current study, we aim to construct a pyroptosis-related gene signature for predicting the prognosis of Wilms’ tumor.

**Methods:**

We acquired RNA-seq data from TARGET kidney tumor projects for constructing a gene signature, and snRNA-seq data from GEO database for validating signature-constructing genes. Pyroptosis-related genes (PRGs) were collected from three online databases. We constructed the gene signature by Lasso Cox regression and then established a nomogram. Underlying mechanisms by which gene signature is related to overall survival states of patients were explored by immune cell infiltration analysis, differential expression analysis, and functional enrichment analysis.

**Results:**

A pyroptosis-related gene signature was constructed with 14 PRGs, which has a moderate to high predicting capacity with 1-, 3-, and 5-year area under the curve (AUC) values of 0.78, 0.80, and 0.83, respectively. A prognosis-predicting nomogram was established by gender, stage, and risk score. Tumor-infiltrating immune cells were quantified by seven algorithms, and the expression of CD8( +) T cells, B cells, Th2 cells, dendritic cells, and type 2 macrophages are positively or negatively correlated with risk score. Two single nuclear RNA-seq samples of different histology were harnessed for validation. The distribution of signature genes was identified in various cell types.

**Conclusions:**

We have established a pyroptosis-related 14-gene signature in WT. Moreover, the inherent roles of immune cells (CD8( +) T cells, B cells, Th2 cells, dendritic cells, and type 2 macrophages), functions of differentially expressed genes (tissue/organ development and intercellular communication), and status of signaling pathways (proteoglycans in cancer, signaling pathways regulating pluripotent of stem cells, and Wnt signaling pathway) have been elucidated, which might be employed as therapeutic targets in the future.

**Supplementary Information:**

The online version contains supplementary material available at 10.1186/s12887-024-04731-0.

## Introduction

Wilms’ tumor (WT) is the most common renal tumor and the second most common malignant abdominal tumor in childhood. The incidence of WT in general population is 0.5–7.5 per million and is lower in high-income areas [1]. Current treatment strategy for WT is based on genetic markers, histology, stage, and other risk factors, which spare children with low-risk tumors from intensive treatment and intensify treatment for children with high-risk tumors [2]. Outcomes and long-term survival have improved over the decades [1, 3].

WTs are divided into two histologies: the favorable histology (FH) and the unfavorable histology (UH) which includes anaplastic histology (AH), clear cell sarcoma of the kidney (CCSK), and malignant rhabdoid tumor (MRT) [4]. Despite the advances in multi-disciplinary treatment and risk-based management of WT, the current prognoses of patients with unfavorable histology remain dismal [5].

Pyroptosis is a type of gasdermin-mediated, inflammation-characterized, and immune-related programmed cell death, which has received increasing attention due to its association with immune response in neoplastic and non-neoplastic diseases [6]. The role of pyroptosis has been extensively elucidated in cardiovascular diseases, nervous system disorders, psychiatric disorders, infection diseases, periodontal diseases, etc. [7–11]. Further, pyroptosis has been proven to be closely related to the biological behavior of multiple tumors [12].

The tumor microenvironment (TME) consists of miscellaneous cell types, such as cancer cells, cancer stem cells, immune cells, stromal cells, and vascular endothelial cells. Tumor-infiltrating immune cells are found generic in tumor tissues with complex tumor-antagonizing or tumor-promoting functions, which surprisingly can affect the hallmarks of the tumor [13]. Quantification methods of these cells are divided into two categories: methods of enrichment on marker genes and methods leveraging the deconvolution algorithm [14, 15]. Each of these methods has a property to allow intra- or inter-sample comparison. We used several authoritative methods in current research.

**Fig. 1:**
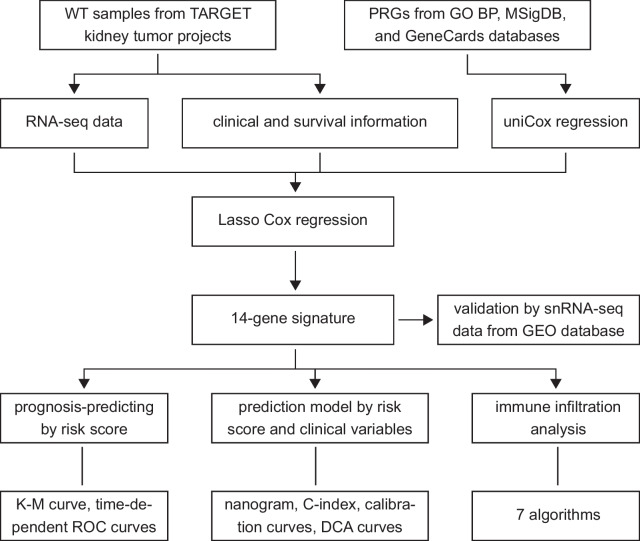
Work flowchart of current study

The RNA-sequencing technique has been extensively employed to identify transcriptome profiles of various tumors [16], based on which several gene signatures have been established for the prognosis of WT [17, 18]. We have previously constructed a ferroptosis-related lncRNA signature in WT [17]. To the best of our knowledge, no signature based on pyroptosis for WT was reported. Here, we established a pyroptosis-related gene signature from the RNA-seq data of WT and constructed a nomogram with the signature and clinical variables. Immune infiltration characteristics of the signature were then identified. We further used snRNA-seq data for validation of our signature. The overall workflow is presented in Fig. 1.

## Material and methods

### Acquisition of gene expression profiles and case information

RNA-seq data (counts value) of 132 tissue samples derived from WT patients were downloaded from Xena datasets (https://xena.ucsc.edu/), which originally came from the TARGET kidney tumor projects (https://ocg.cancer.gov/programs/target) [19]. Of the 132 samples, 126 were from tumor tissues and six were from normal tissues. Supplemental clinical and survival information was acquired from the same source. Preprocessing of expression matrices was completed before further analysis. Briefly, the logarithmed count values downloaded from Xena datasets were transformed by taking anti-logarithms. Genes with counts equal to zero in all the samples were eliminated and a processed counts matrix was obtained, which was used for differential expression analysis. The processed counts data was transformed to transcript per million (TPM) [20], which was used for other subsequent analyses.

### Identification of prognosis-associated pyroptosis-related genes

Pyroptosis-related genes (PRGs) were obtained from three online databases, among which were Gene Ontology Biological Process database (http://geneontology.org/) [21, 22], Molecular Signatures Database (MSigDB) (https://www.gsea-msigdb.org/gsea/index.jsp) [23, 24], and GeneCards database (https://www.genecards.org/) [25]. The union of genes was considered PRGs. The PRGs were then filtered by univariate Cox analysis (uniCox) of overall survival (OS) with R package survival (version 3.5–5, https://github.com/therneau/survival), in which PRGs with *p*-values less than 0.05 were selected as prognosis-associated PRGs.

### Generation of a pyroptosis-related gene signature

The prognosis-associated PRGs were further admitted into Lasso Cox regression analysis with R package glmnet (version 4.1–7, https://glmnet.stanford.edu/) [26]. The function “cv.glmnet” was run for cross-validation and returned a lambda sequence, among which the value of lambda that gave a minimum mean cross-validated error (cvm) was used to extract model coefficients for each PRG. Only PRG with a non-zero coefficient was included in constructing a prognostic gene signature which was then established with eligible PRGs and their coefficients. After, the risk score was calculated for each sample as the following formula: Risk score = Σ Coefficient (PRGs) × Expression (PRGs). Further, samples were divided into high- and low-risk groups by the median risk score. The signature was then verified by plotting Kaplan–Meier (K-M) curve and time-dependent receiver operating characteristic (ROC) curves of 1, 3, and 5 years.

### Construction of a prognosis-predicting model

Univariate Cox regression was applied to risk scores and four clinical variables including gender, age, histology of the tumor, and stage to test the predicting capacity of each variable for OS of patients. Only variables with *p*-values < 0.05 were included into Multivariate Cox regression (multiCox). Multivariate Cox regression method was then applied to these variables, based on which a nomogram was further constructed for the prediction of 1-, 3-, and 5-year overall survival states. And the model was validated by C-index, calibration curves, and decision curve analysis (DCA). Uni- and multi-Cox regression was performed by R package survival (version 3.5–5, https://github.com/therneau/survival) and the nomogram was constructed by R package rms (version 6.7–0, https://hbiostat.org/r/rms/).

### Quantification of tumor-infiltrating immune cells

Three categories of immune cell infiltration analysis were performed among tumor samples. First, the ESTIMATE algorithm was employed to estimate tumor purity with R package estimate (version 1.0.13/r21, https://R-Forge.R-project.org/projects/estimate/) [27]. Second, three marker-gene-based approaches including ssGSEA, xCell, and MCP-counter algorithm were used to calculate enrichment scores of various immune cell types by R packages GSVA (version 1.50.1, https://github.com/rcastelo/GSVA) [28], xCell (version 1.1.0, https://github.com/dviraran/xCell) [29], and MCPcounter (version 1.2.0, https://github.com/ebecht/MCPcounter) [30], respectively. Last, three deconvolution-based approaches which include quanTIseq, CIBERSORT, and CIBERSORT abs. mode were employed to compute relative or absolute fractions of different immune cell types with R packages quantiseqr (version 1.8.0, https://bioconductor.org/packages/quantiseqr) [31] and immunedeconv (version 2.0.3, https://github.com/omnideconv/immunedeconv) [14], respectively. Intra- or inter-sample comparisons were performed in accordance with the characteristic of each algorithm [15].

### Identification of differentially expressed genes and functional enrichment analysis

Differentially expressed genes (DEGs) were identified between high- and low-risk groups by R package limma (version 3.56.2, https://bioinf.wehi.edu.au/limma/) [32]. The Benjamini-Hochberg’s method was used to adjust *p*-values for multiple comparisons [33]. Cut-off threshold was set as |log2FC|> 1 and adjusted *p*-value < 0.05. Function and pathway enrichment analysis of Gene Ontology (GO) database and Kyoto Encyclopedia of Genes and Genomes (KEGG) database were performed by R package clusterProfiler (version 4.8.1, https://www.liebertpub.com/doi/full/10.1089/omi.2011.0118) [34].

### Validation of pyroptosis-related gene signature by snRNA-seq data

Single nuclear RNA-seq (snRNA-seq) data of two WT samples (GSM6025607, and GSM6025616) were gained from GEO database (https://www.ncbi.nlm.nih.gov/geo/). The two snRNA-seq samples are of two different histological types (GSM6025607 was the anaplastic type whereas GSM6025616 was the favorable type) and were analyzed by R package Seurat (version 4.3.0.1, https://github.com/satijalab/seurat) [35]. The number of genes in each cell and the fraction of mitochondrial genes were detected. Cells with unique feature counts of 200–4000 and mitochondrial counts < 5% were sustained. After, the snRNA-seq data was normalized by method “LogNormalize”. Further, principal component analysis (PCA) and t-distributed statistical neighbor embedding (tSNE) were performed to accomplish dimensional reduction. Based on the marker genes, clusters were annotated by results of authoritative literature [36].

### Statistical analysis and data visualization

All statistical analyses were conducted by R (version 4.1.2). Comparative analysis for quantitative data between groups was performed by Wilcoxon rank test and for qualitative data was performed by Chi-square test. Linear correlation analysis between variables was performed by Spearman correlation analysis. *P*-value threshold was set as 0.05. Data visualization processes were conducted with R, except a Venn diagram was processed by Python (version 3.10.2).

## Results

### Prognosis-associated PRGs were identified by uniCox regression

**Fig. 2:**
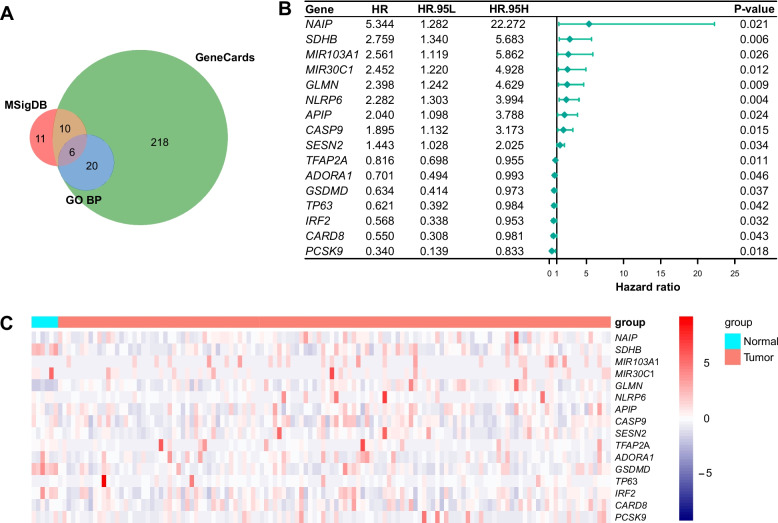
Identification of prognosis-associated PRGs. **A** The Venn diagram of PRGs from Gene Ontology Biological Process database, Molecular Signatures Database, and GeneCards database. **B** The forest plot of prognosis-associated PRGs. **C** The heatmap of gene expression levels (Z-score scaled) of prognosis-associated PRGs in tumor and normal tissues. HR, hazard ratio; HR.95L, lower 95% hazard ratio; HR.95H, higher 95% hazard ratio

A union gene list of 265 genes was exhibited in Fig. 2A and Table S1, among which were not only protein-coding genes but also genes that do not code for proteins. All of them were included in univariate Cox analysis (Table S2), after which 16 genes were kept for further research. The hazard ratio (HR) and *p*-value of each gene were presented in Fig. 2B. The differential expression levels of these genes between tumors and normal tissues were exhibited in Fig. 2C.

### A Pyroptosis-related gene signature was constructed by Lasso Cox regression

**Fig. 3:**
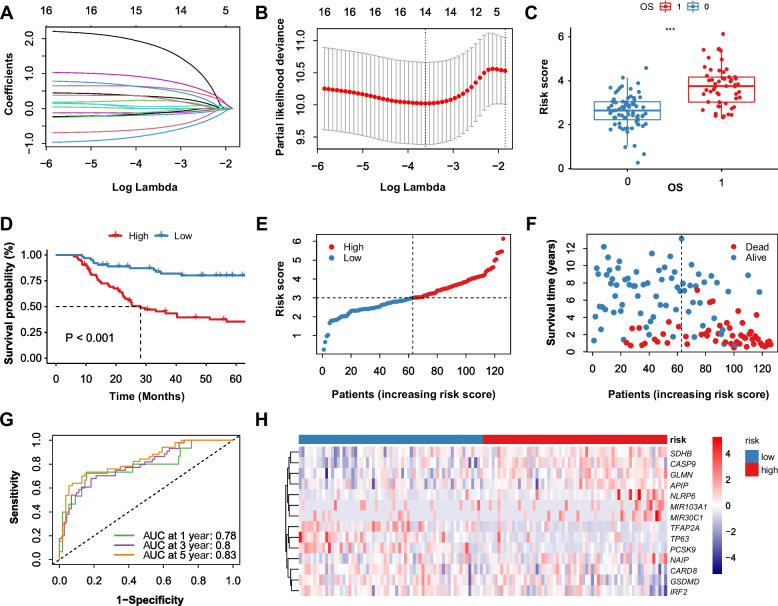
Generation of a pyroptosis-related gene signature. **A**, **B** The lasso Cox regression profile of 14 prognosis-associated PRGs. **C** The distribution of risk scores between OS states of patients. **D** The K-M curve between high- and low-risk groups. **E**, **F** The distribution of risk scores and OS states among patients. **G** The time-dependent ROC curves at 1, 3, and 5 years. **H** The heatmap of gene expression levels (Z-score scaled) of prognosis-associated PRGs between high- and low-risk groups. OS, overall survival; ROC, receiver operating characteristic; AUC, area under the curve

The model-fitting and cross-validation processes were exhibited in Fig. 3A, B. The selected value of lambda that gave a minimum mean cross-validated error (cvm) was marked by the left dotted line in Fig. 3B. 16 candidate PRGs were analyzed by Lasso Cox regression and 14 PRGs were eligible to construct a prognostic gene signature. Signature-constructing genes and their coefficients were listed in Table 1. Among tumor patients, the correlations between risk scores and OS states were presented in Fig. 3C. K-M curves showed that patients in the low-risk group have better survival states than patients in the high-risk group (Fig. 3D).

**Table 1:** 14 signature-constructing genes

Gene symbol	Gene type	Coefficient
*NAIP*	protein-coding	1.810846993
*NLRP6*	protein-coding	0.859953893
*SDHB*	protein-coding	0.61105682
*MIR30C1*	miRNA	0.581803409
*APIP*	protein-coding	0.344031843
*CASP9*	protein-coding	0.317758755
*MIR103A1*	miRNA	0.231369542
*GLMN*	protein-coding	0.061898532
*CARD8*	protein-coding	-0.097182671
*GSDMD*	protein-coding	-0.119052922
*TP63*	protein-coding	-0.137417683
*TFAP2A*	protein-coding	-0.181394255
*IRF2*	protein-coding	-0.566583108
*PCSK9*	protein-coding	-0.736539164

The distribution of risk scores, OS states, and signature genes’ expression was presented in Fig. 3E-G. To confirm the independent predicting power of the gene signature, time ROC curves were plotted (Fig. 3H). And the signature was proved to have a moderate to high predicting capacity with 1-, 3-, and 5-year area under the curve (AUC) values of 0.78, 0.80, and 0.83, respectively.

### A prognosis-predicting model was constructed with 3 variables

**Fig. 4:**
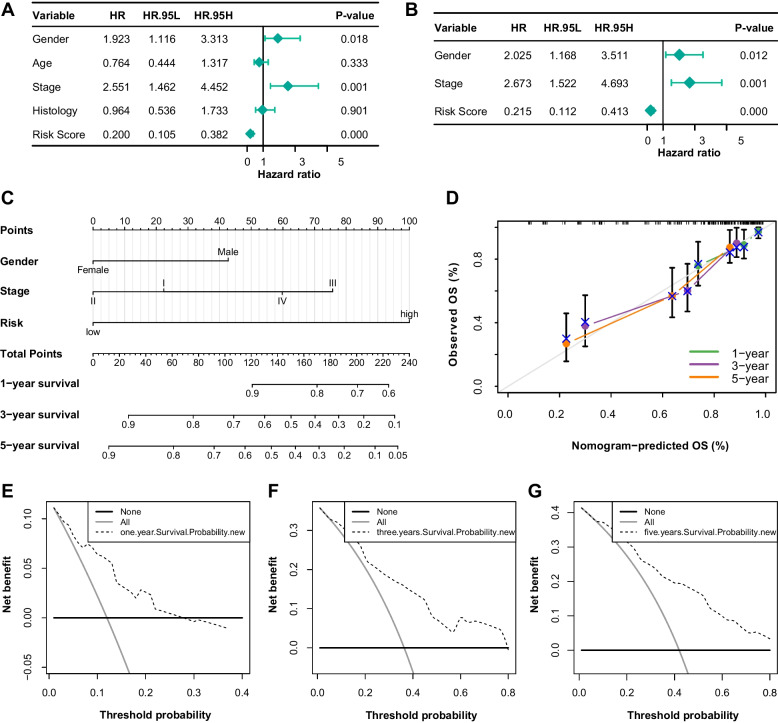
Construction of a prognosis-predicting model. **A**, **B** The forest plots of uni- and multi-Cox regression for risk score and clinical variables. **C** The nomogram is constructed by gender, stage, and risk score. **D** The calibration plot at 1, 3, and 5 years. **E**–**G** The DCA curves at 1, 3, and 5 years. HR, hazard ratio; HR.95L, lower 95% hazard ratio; HR.95H, higher 95% hazard ratio; OS, overall survival; DCA, decision curve analysis

Despite our best efforts, only four clinical variables with high-quality information were extracted, including gender, age, histology of the tumor, and stage (Table 2). Univariate Cox regression analysis was employed to risk score and four clinical variables (Fig. 4A, Table S3). Gender, stage, and risk score were further included into multivariate Cox regression analysis (Fig. 4B, Table S4). A nomogram based on multiCox was exhibited in Fig. 4C. The C-index was 0.758, which indicated a moderate predicting efficiency of the model. The model showed good calibration with the diagonal (Fig. 4D). Moreover, DCA curves showed good benefits in prediction (Fig. 4E-G).

**Table 2:** Clinical information of patients between high- and low-risk groups

	**Overall (126)**	**High-risk (63)**	**Low-risk (63)**	***P* value**
**Risk score (median [IQR])**				
	3.00 [2.42, 3.73]	3.74 [3.33, 4.15]	2.42 [2.04, 2.74]	< 0.001
**Gender (%)**				
Female	70 (55.6)	33 (52.4)	37 (58.7)	0.591
Male	56 (44.4)	30 (47.6)	26 (41.3)	
**Age (median [IQR])**				
	4.30 [2.82, 6.07]	4.50 [3.40, 6.00]	4.00 [2.25, 6.05]	0.307
**Histologic classification (%)**				
DAWT	40 (31.7)	23 (36.5)	17 (27.0)	0.339
FHWT	86 (68.3)	40 (63.5)	46 (73.0)	
**Stage (%)**				
I	16 (12.7)	8 (12.7)	8 (12.7)	0.663
II	52 (41.3)	23 (36.5)	29 (46.0)	
III	45 (35.7)	24 (38.1)	21 (33.3)	
IV	13 (10.3)	8 (12.7)	5 (7.9)	
**Vital status (%)**				
Alive	73 (57.9)	22 (34.9)	51 (81.0)	< 0.001
Dead	53 (42.1)	41 (65.1)	12 (19.0)	

*IQR* interquartile range, *DAWT* diffuse anaplasia Wilms’ tumor, *FHWT* favorable histology Wilms’ tumor

### Tumor-infiltrating immune cells were quantified by multiple algorithms

**Fig. 5:**
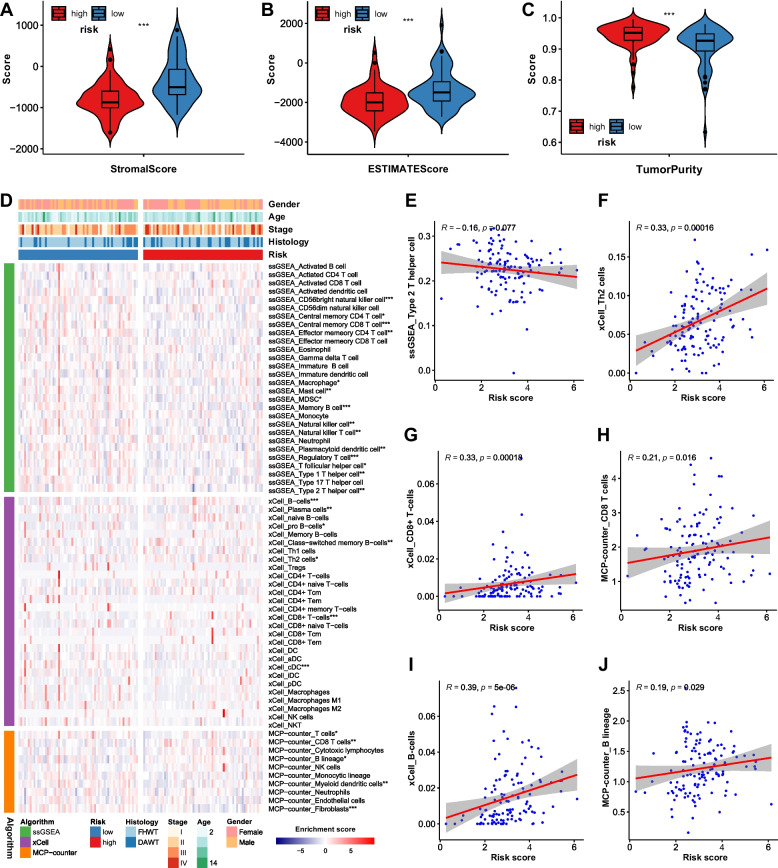
Estimation of tumor purity and quantification of tumor-infiltrating immune cells by marker-gene-based approaches. **A** The estimation of Stromal Scores, ESTIMATE Scores, and Tumor Purity of tumors. **D** The heatmap of enrichment scores (ES) of immune infiltrating cells by marker-gene-based approaches. **E**-**J** The correlation plots of ES of Th2 cells, CD8 + T cells, and B cells with risk scores

Various infiltrating immune cells exert complex functions and impact on biological behavior of tumors. Stromal cells are also thought to be significant for tumor growth [13]. Stromal Score, Immune Score, ESTIMATE Score, and Tumor Purity of each sample were evaluated. The Stromal Scores were significantly higher in low-risk group than those in high-risk group (Fig. 5A). However, no statistical difference was sighted in the Immune Scores (Figure S1A). The ESTIMATE Scores and Tumor Purities indicated that tumors in high-risk group had higher tumor purity (Fig. 5B, C), which might be dominantly caused by Stromal Scores.

The results of marker-gene-based immune cells infiltration analyses were exhibited in heatmap (Fig. 5D). The enrichment scores (ES) of three immune cell types (Th2 cells, CD8( +) T cells, and B cells) were statistically significant between groups in more than one algorithm. The correlation between ES of these three cell types and risk score is shown in Fig. 5E-J. Except for Th2 cells in ssGSEA algorithm, 5 scatter plots showed a low or negligible positive correlation between ES and risk score.

**Fig. 6:**
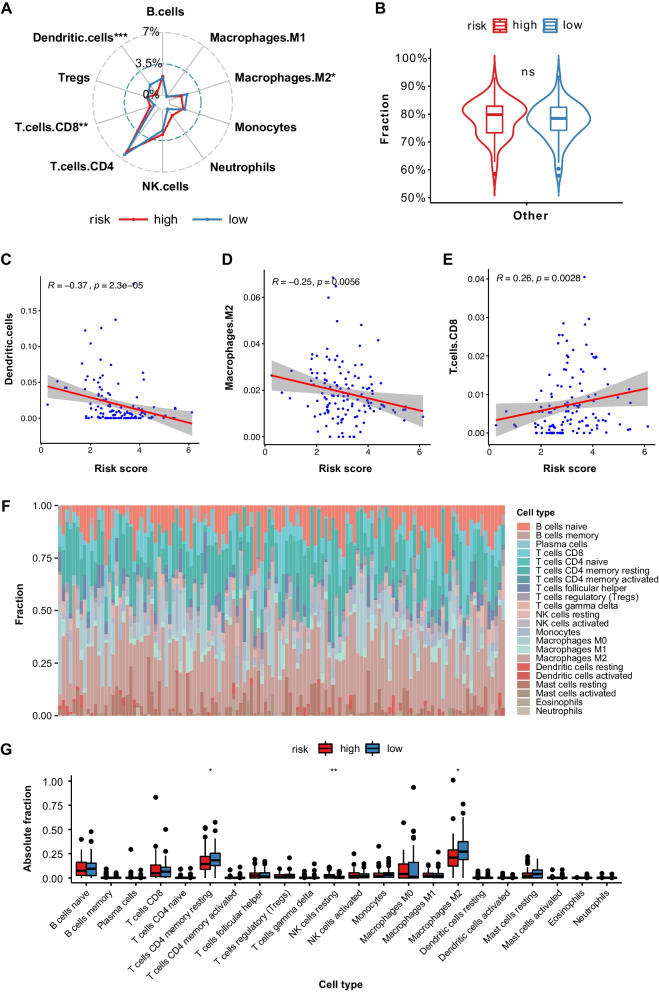
Quantification of tumor-infiltrating immune cells by deconvolution-based approaches. **A**, **B** The differences of absolute fractions of immune infiltrating cells between high- and low-risk groups by quanTIseq algorithm. **C**-**E** The correlation plots of absolute fractions of dendritic cells, macrophages type 2, and CD8 + T cells with risk score. **F** The fraction of each type of immune infiltrating cells in all immune cells by CIBERSORT. **G** The estimated absolute fractions of immune infiltrating cells. *P value < 0.05, **P value < 0.01, ***P value < 0.001

The absolute fractions of immune infiltrating cells were quantified by quanTIseq algorithm (Fig. 6A, B). Immune cells took up a minority of tumor tissue, while “other” cells occupied the majority. Among the immune cell types, CD4 + T cells had a predominantly quantity advantage over other immune cells, while macrophages type 1 had a minute quantity in both groups. Between high- and low-risk groups, three types of immune cells (dendritic cells, macrophages type 2, and CD8( +) T cells) were statistically different in absolute fractions. The correlations of fractions of these three cell types with risk score were shown in Fig. 6C-E, of which CD8( +) T cells had a low or negligible positive correlation with risk score, while macrophages type 2 and dendritic cells had a low or negligible negative correlation with risk score. The correlations among cell types in quanTIseq algorithm were exhibited in Figure S1B.

The fraction of each type of immune infiltrating cells in all immune cells was evaluated by CIBERSORT, as is shown in Fig. 6F. Further, CIBERSORT abs. mode was applied and estimated absolute fractions of each immune cell type were exhibited in Fig. 6G, in which the fraction differences of three immune cell types (resting CD4 + T memory cells, resting NK cells, and macrophages type 2) were statistically significant.

### Differential expression and functional enrichment analysis

**Fig. 7:**
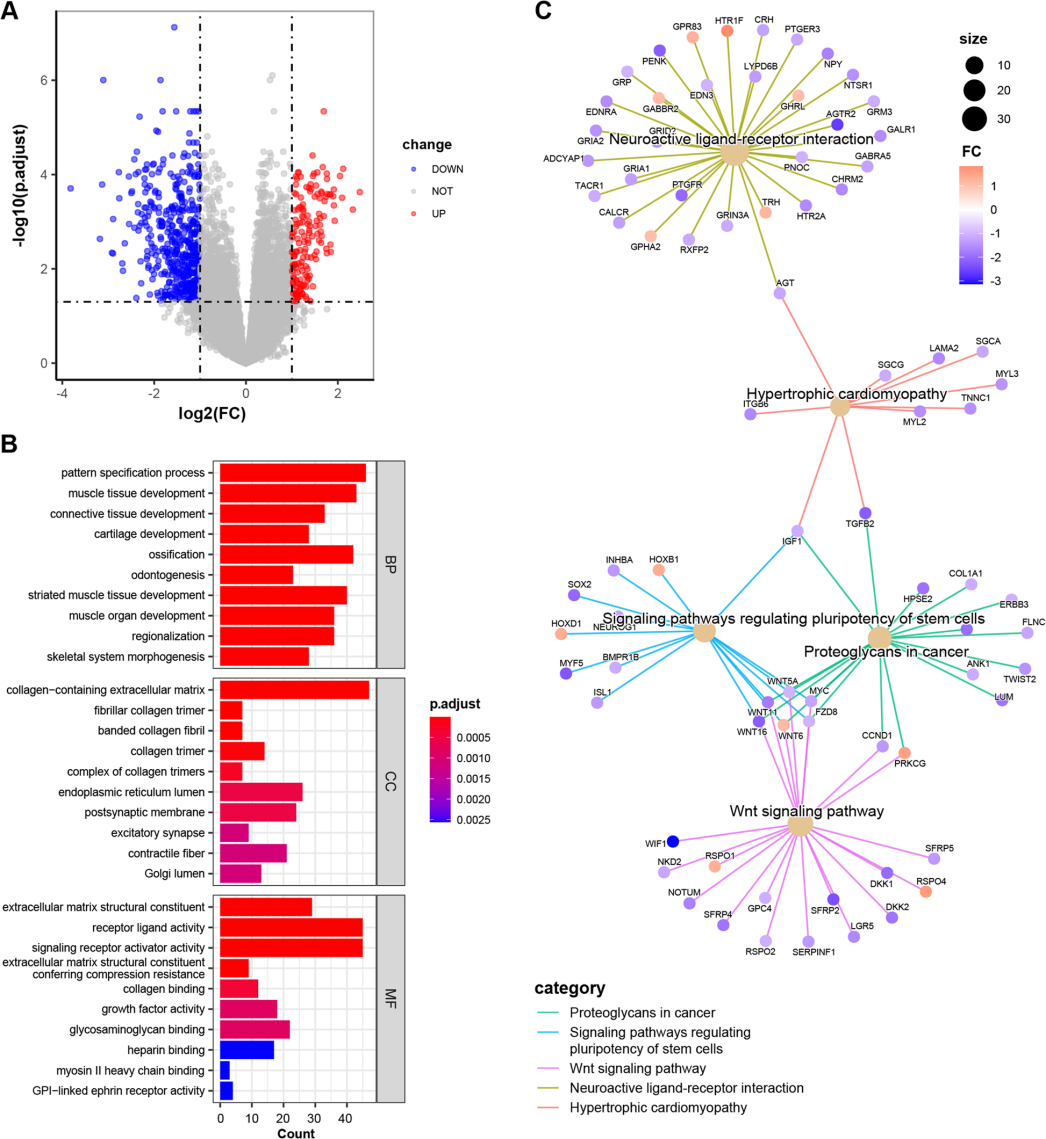
Differential expression and functional enrichment analysis between high- and low-risk groups. **A** The valcano plot of DEGs. **B**, **C** Function and pathway enrichment analysis of GO and KEGG database. GO, gene ontology; KEGG, Kyoto encyclopedia of genes and genomes. p.adjust, adjusted *p*-value; FC, fold change

DEGs between the two groups were visualized in Fig. 7A. Results of functional enrichment analyses for GO and KEGG databases for DEGs were shown in Fig. 7B, C. Overall, enrichment for biological process (BP), cellular component (CC), and molecular function (MF) mainly focused on tissue/organ development and intercellular communication. Of note, enrichment for pathways in KEGG database indicated that DEGs were mainly enriched in proteoglycans in cancer, signaling pathways regulating pluripotent of stem cells, and Wnt signaling pathway, which were highly related to tumor invasion and metastasis [37–43].

### Validation of pyroptosis-related gene signature by snRNA-seq data

**Fig. 8:**
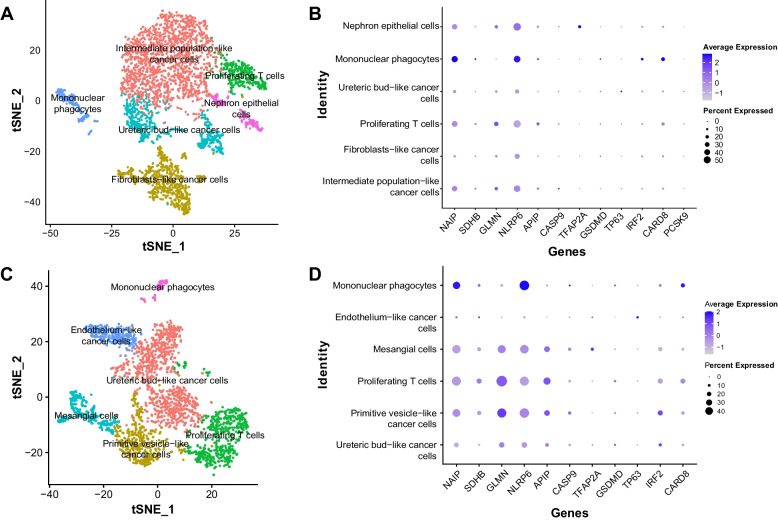
Validation of pyroptosis-related gene signature by snRNA-seq data. **A**, **B** The annotation of clusters and distribution of signature genes in the sample of favorable histology. **C**, **D** The annotation of clusters and distribution of signature genes in the sample of anaplastic histology. tSNE, t-distributed stochastic neighbor embedding

Favorable histology and anaplastic histology are two distinct groups of WT. The snRNA-seq data of samples from each histologic type were analyzed respectively. Consistent with the annotation method of an authoritative research, clusters of the two samples were annotated in Fig. 8A, C. The sample of favorable histology had clusters of intermediate population-like cancer cells, ureteric bud-like cancer cells, fibroblasts-like cancer cells, nephron epithelial cells, proliferating T cells, and mononuclear phagocytes. The sample of anaplastic histology had clusters of ureteric bud-like cancer cells, primitive vesicle-like cancer cells, endothelium-like cancer cells, mesangial cells, proliferating T cells, and mononuclear phagocytes.

Of the 14 signature genes, *NAIP* and *TP63* were considered marker genes for mononuclear phagocytes and ureteric bud-like cancer cells respectively in the sample of favorable histology. In the sample of anaplastic histology, *GLMN* and *NAIP* were considered marker genes for primitive vesicle-like cancer cells and endothelium-like cancer cells, respectively.

The distribution of 14 signature genes in each cell type and all cells was exhibited in Fig. 8B, D, and Figure S1C, D. The signature genes’ expression was relatively low in ureteric bud-like cancer cells, fibroblasts-like cancer cells, and endothelium-like cancer cells in the two samples. Of note, *NLRP6* and *NAIP* had the highest expression while *GSDMD* and *TP63* had the lowest expression in both the samples.

## Discussion

Wilms’ tumor is an embryonal malignancy derived from nephrogenic blastemal cells in nephrogenic rests [2]. Current treatment strategy for WT from Children’s Oncology Group (COG) is based on traditional risk factors, including histology, stage, age, tumor weight, response to therapy, and loss of heterozygosity at 1p and 16q [44]. These prognostic factors are derived from elaborate clinical trials and have been guiding operative therapy, chemotherapy, and radiotherapy of WT. Here, we have screened out gender and stage as prognostic clinical factors in WT. To acquire a more precise risk-classifying approach, exploring specific patterns of gene expression profiles in tumor tissue is necessary.

Characterized by inflammation, pyroptosis is a more intense pattern of cell death compared with apoptosis, leading to pore formation on cell membrane, chromatin fragmentation, cell swelling, and osmotic lysis of the cell [6]. Exact molecular relationship between pyroptosis and tumors’ bio-behavior remains unclear, but studies have found that enhancement of pyroptosis in tumors leads to inhibition of tumor progression [45–49]. To the best of our knowledge, few studies have elucidated the role of pyroptosis in WT.

In this study, we have established a pyroptosis-associated gene signature with 14 PRGs, among which *CARD8*, *GSDMD*, *TP63*, *TFAP2A*, *IRF2*, and *PCSK9* were negatively correlated with risk score, while *NAIP*, *NLRP6*, *SDHB*, *MIR30C1*, *APIP*, *CASP9*, *MIR103A1*, and *GLMN* were positively correlated with risk score.

Caspase recruitment domain family member 8 (*CARD8*) is an inflammasome sensor, which ultimately activates *GSDMD* and inflammatory cytokines, leading to pyroptosis [50]. Gasdermin D (*GSDMD*) is the main executioner of pyroptosis, and is considered as a tumor suppressor [51, 52]. It has been deeply studied in pyroptotic cell death. Transcription factor AP-2 alpha (*TFAP2A*) and interferon regulatory factor 2 (*IRF2*) were proven to transcriptionally induce *GSDMD* by binding to its promoter, which subsequently induces pyroptosis [53, 54]. The role of proprotein convertase subtilisin/kexin type 9 (*PCSK9*) in pyroptosis has been clarified in cardiomyocytes and vascular endothelial cells [55, 56]. These five genes are pyroptosis-executive or pyroptosis-promoting, and in the sight of the tumor-suppressing role of pyroptosis, these are reasonable to be negatively correlated with risk score.

APAF1 interacting protein (*APIP*) is validated to inhibit pyroptosis and apoptosis [57, 58]. Glomulin, FKBP Associated Protein (*GLMN*) is a negative regulator of pyroptosis via regulating cIAP-mediated inflammasome activation [59, 60]. These two genes have the pyroptosis-inhibiting effect, which is easy to explain the positive correlations with risk score.

NLR family apoptosis inhibitory protein (*NAIP*) is necessary for inflammasome assembly which subsequently cleaves caspase-1 and leads to pyroptosis [61]. Like *CARD8*, NLR family pyrin domain containing 6 (*NLRP6*) is also an inflammasome sensor that mediates inflammasome activation and promotes recruitment of effector proinflammatory caspases [62]. Overexpression of succinate dehydrogenase complex iron sulfur subunit B (*SDHB*) has been proven to enhance pyroptosis in vascular endothelial cells [63]. Caspase 9 (*CASP9*) has the ability to cleave and activate caspase-3, which subsequently activates Gasdermin E (*GSDME*) [64]. These four genes are pyroptosis-executive or pyroptosis-promoting genes. Providing the negative correlation of pyroptosis with tumor progression, the four genes are supposed to be negatively correlated with risk score, which contradicts our observations.

Here are possible explanations. First, the predominant role of pyroptosis in the bio-behavior of tumors is tumor suppressor, but the adverse effect may exist in certain tumor microenvironments. Second, except for pyroptosis, the functions of genes are most likely to relate to multiple biological processes. Such as *NAIP* can also act as an anti-apoptotic protein by inhibiting caspase-3, and caspase-7 [65, 66], which may promote tumorigenesis and tumor invasion. Third, few studies have explored the exact roles of certain genes (*NLRP6 and SDHB*) in WT. Although *NLRP6* was reported to serve as a tumor suppressor in colorectal cancer, hepatocellular carcinoma, and gastric cancer [67–69], it was reported to restore immune evasion and radio-resistance in glioma through ASC/caspase-1/IL-1β axis [70]. The *SDHB* gene encodes the iron-sulfur protein subunit of the succinate dehydrogenase enzyme complex which plays a critical role in respiratory electron transport and tricarboxylic acid cycle [71]. Higher gene expression of *SDHB* may provide more energy for tumor cells in WT. The roles of these genes in WT need further investigation. Last but not the least, genes may have complex interactions with the tumor microenvironment. For example, *CASP9* inhibition triggers immunogenic cell death, increases tumor-intrinsic innate sensing, and induces remarkable anti-tumor effects in chemotherapy-induced anti-tumor immunity [72]. Higher gene expression of *CASP9* may exhibit less immunogenic cell death and worse prognosis in WT. The above-mentioned theoretical deductions need further experiment-based validation.

Tumor protein p63 (*TP63*) has two isoforms, in which TAp63 is thought of as a tumor suppressor, while ΔNp63 is considered as an oncogene [73]. In the sample set of our study, TAp63 may be functionally predominant over ΔNp63. MicroRNA 30c-1 (*MIR30C1*) is known to inhibit the progression of prostate cancer and the invasion of melanoma [74, 75]. MicroRNA 103a-1 (*MIR103A1*) was found to have dual effects on different tumor types [76–78]. In our results of bioinformatics analysis, *MIR103A1* is most likely to negatively regulate the progression of WT.

Solid tumor tissue does not only have cancer cells but also immune inflammatory cells, endothelial cells, etc. Tumor-promoting and tumor-antagonizing immune cells exist simultaneously in tumors [13]. In our observations of immune cell infiltration, the expression of CD8( +) T cells, B cells, and Th2 cells are positively correlated with risk score, while the expression of dendritic cells and type 2 macrophages are negatively correlated with risk score.

Dendritic cells can either suppress tumor progression or drive tolerance in the TME [79]. As for our results, dendritic cells are most likely to play a tumor suppressor role in WT. B cells are considered to have dual effects in the TME, and Th2 cells are the intrinsic helper for B cells [80]. They are both positively related to risk score in our research, based on which we can assume that the tumor-promoting effect overweighs the tumor-suppressing effect.

CD8( +) T cells, also called cytotoxic lymphocytes (CTLs), have long been known to relate to better survival in tumors [81–83]. But in our results, it is positively related to risk score. Type 2 macrophages are known to be tumor-promoting [84, 85], which contradicts our observations, too. There are several explanations. First, it is the enrichment scores or fractions of immune cells that stand for expression levels, but not the real quantity of them. Second, despite our best efforts, only one dataset in TARGET database was included in our research, and no validating dataset of good quality was harnessed. Thereby, the sample number of tumors is only 126, which may be too small to get completely correct results. Third, it may be reasonable to assume that the more invasive the tumor is, the more activation of immune system to fight against it. Based on this assumption, the role of immune infiltrating cells in WT is more of a defensive reactor than a prognostic indicator.

Hence, the distinct immune cells infiltration landscape between high- and low-risk groups was mainly made up of CD8( +) T cells, B cells, Th2 cells, dendritic cells, and type 2 macrophages, which were validated by various algorithms.

WT classically consists of three elements: blastemal, stromal, and epithelial tubules, which are normal components of developing kidneys. Less commonly, skeletal muscle, cartilage, osteoid, or adipose tissue can also be found in WT [2, 4]. In our study, GO BP analysis showed DEGs between two groups were mainly enriched in tissue/organ development, the top five of which were pattern specification process, muscle tissue development, connective tissue development, cartilage development, and ossification. These observation indicates that the extent of development of embryonal components in WT is correlated with tumor progression and prognostic risk. KEGG analysis showed pathway enrichment in signaling pathways regulating pluripotency of stem cells, proteoglycans in cancer, and Wnt signaling pathway. WT has similar components to fetal kidneys, where pluripotent stem cells (PSCs) play a key role in tissue formation and organ development. Our results suggest the signaling pathway that regulates PSCs is correlated with tumorigenesis of WT. Proteoglycans (PGs) and the Wnt signaling pathway have been extensively studied in multiple cancers, both of which are closely related to tumor progress and invasion [37–43].

Therefore, the extent of development of embryonal components in WT together with the status of proteoglycans and Wnt signaling pathway might be intermediate contributors to tumor progression.

Single-cell RNA-seq (scRNA-seq) and single-nucleus RNA-seq (snRNA-seq) have received increasing attention over the years. Both techniques have remarkable advantages over bulk RNA-seq. We have employed a recently published snRNA-seq dataset of WT to validate our gene signature. In accordance with a former authoritative study [36], cell clusters are similar to normal constituents in fetal kidneys, which is a distinct characteristic of WT. Combined with the results of GO/KEGG analysis, we might infer that the capacity of tumor cells to generate various renal cells isn’t lost or is required in WT.

Thus, the snRNA-seq dataset has facilitated the validation of signature-establishing genes and has supported the renal lineage-generating ability of tumor cells in WT.

The tumor microenvironment has complex gene interactions and cell cross-talks, which form latent networks. Identifying key modules and hub genes by weighted gene co-expression network analysis might provide a deeper understanding of the roles of signature-constructing genes and tumor-infiltrating immune cells like another study [86], which needs future exploration.

Several limitations are supposed to be acknowledged. First, as mentioned before, only one bulk RNA-seq dataset of good quality was used in our research, which might not draw completely correct conclusions. Second, due to the particularities of constituents of WT, the annotation process for each cluster in snRNA-seq analysis was mainly based on one literature of authority, which might lack rigor. Last, compared with clinical factors, there is still a long way to go to perform risk classification based on tumor transcriptome.

The current study is mainly based on retrospective data analysis and in silico experiments. Further validation is essential to confirm the exact roles of signature-constructing genes and tumor-infiltrating immune cells. For future investigation, we aim to perform gene-overexpression or knockdown in WT cell lines and observe changes in proliferation, migration, invasion, and other malignant behavior in vitro and in vivo. Moreover, a long-term follow-up of patient cohorts grouped by the gene signature will provide validation for the current study.

## Conclusions

Here, we have established a pyroptosis-related gene signature of moderate to high predicting capacity with 14 PRGs and have constructed a prognostic-predicting nomogram with risk score and two clinical variables. Moreover, immune cell infiltration analysis, differential expression analysis, and functional enrichment analysis were performed to further explore underlying mechanisms contributing to the differences in survival states. Our research provides insights into the role of pyroptosis and possible therapeutic targets in Wilms’ tumor.

### Supplementary Information

**Additional file 1: Figure S1.** (A) The estimation of Immune Scores of tumors. (B) The correlation heatmap of immune infiltrating cells by quanTIseq algorithm. (C-D) The distribution of signature genes in each cluster in the samples of favorable histology and anaplastic histology, respectively. tSNE, t-distributed stochastic neighbor embedding.

**Additional file 2: Table S1.** The list of PRGs from three online databases. **Table S2.** The prognostic capacities of PRGs by univariate Cox regression. **Table S3.** The prognostic capacities of risk score and clinical variables by univariate Cox regression. **Table S4.** The prognostic capacities of risk score and clinical variables by multivariate Cox regression.

## Data Availability

The datasets analyzed during the current study are available in the Xena datasets (https://xena.ucsc.edu/) and GEO database (https://www.ncbi.nlm.nih.gov/geo/).

## Abbreviations

WT: Wilms’ tumor
PRGs: Pyroptosis-related genes
AUC: Area under the curve
FH: Favorable histology
UH: Unfavorable histology
AH: Anaplastic histology
CCSK: Clear cell sarcoma of the kidney
MRT: Malignant rhabdoid tumor
TME: Tumor microenvironment
snRNA-seq: Single nuclear RNA-seq
MSigDB: Molecular Signatures Database
OS: Overall survival
K-M: Kaplan–Meier
ROC: Receiver operating characteristic
DCA: Decision curve analysis
DEGs: Differentially expressed genes
GO: Gene Ontology
KEGG: Kyoto Encyclopedia of Genes and Genomes
PCA: Principal component analysis
tSNE: T-distributed statistical neighbor embedding
HR: Hazard ratio
ES: Enrichment score
BP: Biological process
CC: Cellular component
MF: Molecular function
COG: Children’s Oncology Group
CARD8: Caspase recruitment domain family member 8
GSDMD: Gasdermin D
TFAP2A: Transcription factor AP-2 alpha
IRF2: Interferon regulatory factor 2
PCSK9: Proprotein convertase subtilisin/kexin type 9
APIP: APAF1 interacting protein
GLMN: Glomulin, FKBP Associated Protein
NAIP: NLR family apoptosis inhibitory protein
NLRP6: NLR family pyrin domain containing 6
SDHB: Succinate dehydrogenase complex iron sulfur subunit B
CASP9: Caspase 9
GSDME: Gasdermin E
TP63: Tumor protein p63
MIR30C1: MicroRNA 30c-1
MIR103A1: MicroRNA 103a-1
CTLs: Cytotoxic lymphocytes
PSCs: Pluripotent stem cells
PGs: Proteoglycans
scRNA-seq: Single-cell RNA-seq
DAWT: Diffuse anaplasia Wilms’ tumor
FHWT: Favorable histology Wilms’ tumor
