# Supplementary figures and images for: A novel pyroptosis-related gene signature exhibits distinct immune cells infiltration landscape in Wilms’ tumor
Source: BMC Pediatr. 2024 Apr 27;24:279. doi: 10.1186/s12887-024-04731-0 (PMC11055250; doi:10.1186/s12887-024-04731-0)

**A**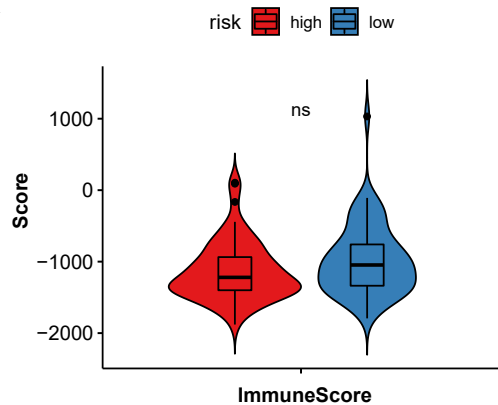**B**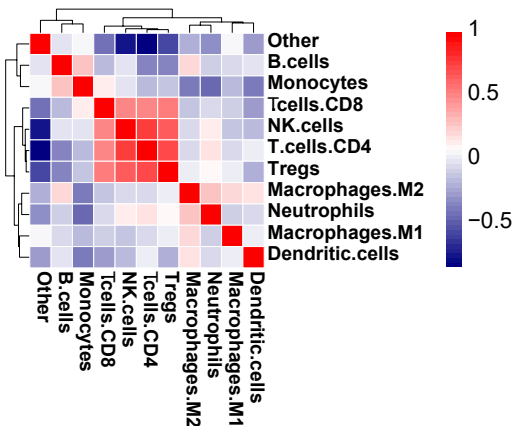**C**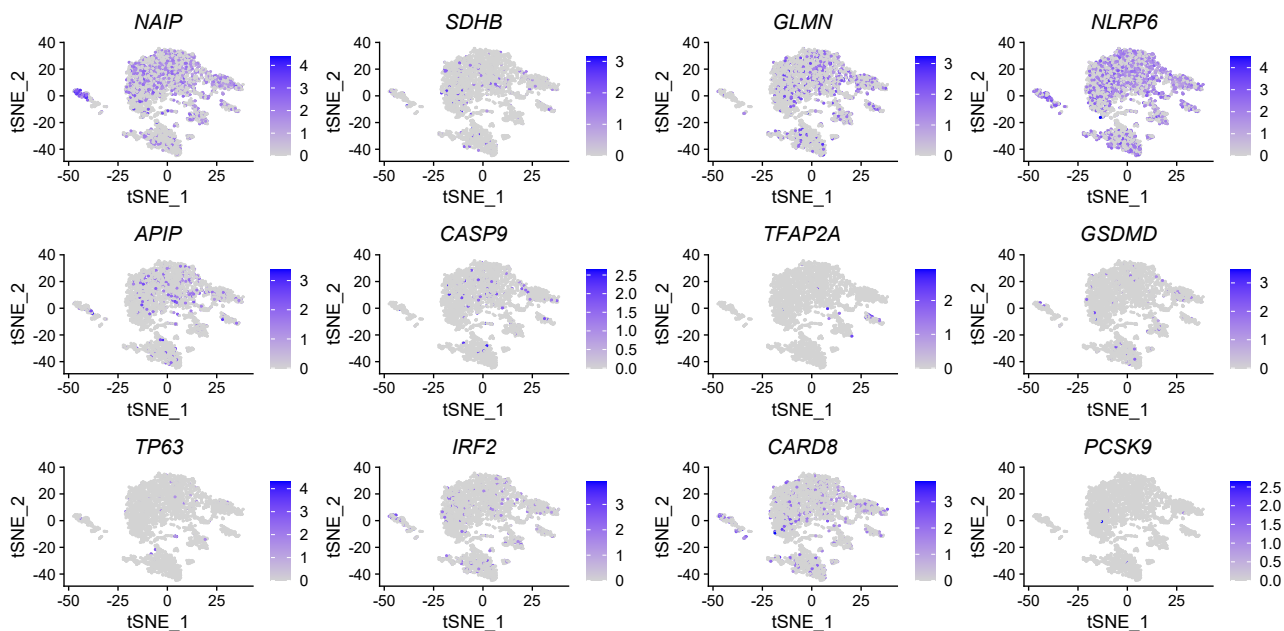**D**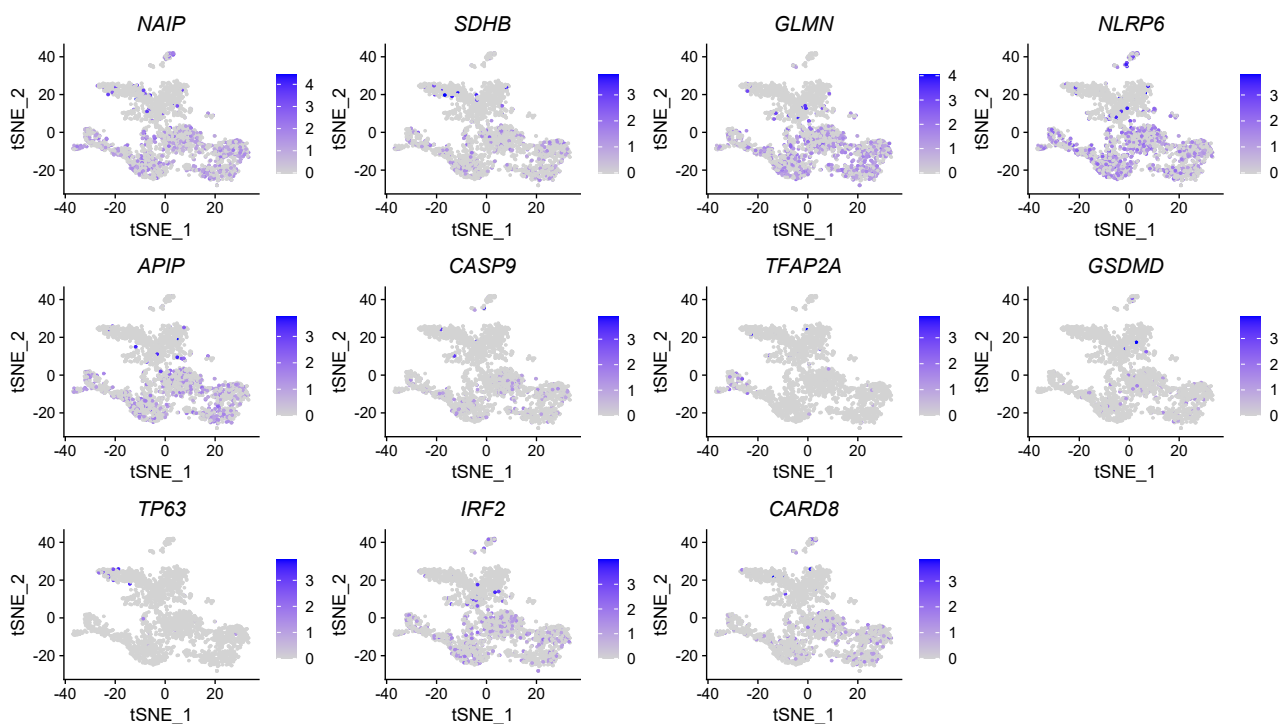

Supplement: Supplementary file 1 — Additional file 1: Figure S1. (A) The estimation of Immune Scores of tumors. (B) The correlation heatmap of immune infiltrating cells by quanTIseq algorithm. (C-D) The distribution of signature genes in each cluster in the samples of favorable histology and anaplastic histology, respectively. tSNE, t-distributed stochastic neighbor embedding. [file 12887_2024_4731_MOESM1_ESM.pdf]
